# The analgesic efficacy and safety of peri-articular injection versus intra-articular injection in one-stage bilateral total knee arthroplasty: a randomized controlled trial

**DOI:** 10.1186/s12871-019-0922-4

**Published:** 2020-01-04

**Authors:** Kai-Yuan Cheng, Bin Feng, Hui-Ming Peng, Yan-Yan Bian, Lin-Jie Zhang, Chang Han, Gui-Xing Qiu, Xisheng Weng

**Affiliations:** 1 the Department of Orthopaedic Surgery, Peking Union Medical College Hospital, Beijing, 100730 China; 2 Chinese Academy of Medical Sciences and Peking Union Medical College, Beijing, 100730 China

**Keywords:** Peri-articular injection, Intra-articular injection, Total knee arthroplasty, Pain management

## Abstract

**Background:**

As an essential component of multimodal analgesia approaches after total knee arthroplasty (TKA), local infiltration analgesia (LIA) can be classified into peri-articular injection (PAI) and intra-articular injection (IAI) according to administration techniques. Currently, there is no definite answer to the optimal choice between the two techniques. Our study aims to investigate analgesic efficacy and safety of PAI versus IAI in patients receiving simultaneous bilateral TKA.

**Methods:**

This randomized controlled trial was conducted from February 2017 and finished in July 2018. Sixty patients eligible for simultaneous bilateral total knee arthroplasty were randomly assigned to receive PAI on one side and IAI on another. Primary outcomes included numerical rating scale (NRS) pain score at rest or during activity at 3 h, 6 h, 12 h, 24 h, 48 h, and 72 h following surgery. Secondary outcomes contained active or passive range of motion (ROM) at 1, 2, and 3 days after surgery, time to perform straight leg raise, wound drainage, operation time, and wound complications.

**Results:**

Patients experienced lower NRS pain scores of the knee receiving PAI compared with that with PAI during the first 48 h after surgery. The largest difference of NRS pain score at rest occurred at 48 h (PAI: 0.68, 95%CI[0.37, 0.98]; IAI: 2.63, 95%CI [2.16, 3.09]; *P* < 0.001); and the largest difference of NRS pain score during activity also took place at 48 h (PAI: 2.46, 95%CI [2.07, 2.85]; IAI: 3.90, 95%CI [3.27, 4.52]; *P* = 0.001). PAI group had better results of range of motion and time to perform straight leg raise when compared with IAI group. There were no differences in operation time, wound drainage, and wound complication.

**Conclusion:**

PAI had the superior performance of pain relief and improvement of range of motion to IAI. Therefore, the administration technique of peri-articular injection is recommended when performing local infiltration analgesia after total knee arthroplasty.

**Trial registration:**

The trial was retrospectively registered in the Chinese Clinical Trial Registry as ChiCTR1800020420 on 29th December, 2018.

**Level of evidence:**

Therapeutic Level I.

## Background

Although total knee arthroplasty (TKA) has been recognized as the optimal treatment method for the end stage of knee osteoarthritis, over 50% patients experienced moderate to severe postoperative pain after receiving the surgery [1]. Perioperative pain management in TKA may be insufficient and hinders the process of fast recovery [2]. Multimodal analgesia regimen gains popularity in recent years, encompassing patient-controlled analgesia [3], epidural analgesia [4], femoral nerve block [5], and local infiltration analgesia [6]. However, every single method has its pros and cons: patient-controlled analgesia (PCA) is quite useful for severe pain, but it could also result in sequent side effects such as nausea, vomiting, constipation, and respiratory depression [7]; the epidural analgesia involving intrathecal injection raised the risk of nausea, hypotension, and respiratory depression [8]; despite adequate analgesia of femoral nerve block, it has been associated with quadriceps weakness and increased risk of in-hospital falls [9]. In recent years, local infiltration analgesia (LIA) is becoming more commonly applied in TKA for its convenience, splendid analgesic efficacy, and fewer side effects [10–12].

LIA is commonly performed as direct injection of a cocktail solution containing local anaesthetic, opioids, adrenaline, glucocorticoids, and nonsteroidal anti-inflammatory drugs (NSAIDs) into the surgical area to relieve inflammation and pain [13, 14]. Administration techniques of LIA could be classified into peri-articular injection (PAI) and intra-articular injection (IAI). It is well-known that exogenous IAI of hyaluronate is valid as a treatment for the symptoms of knee osteoarthritis [15]. IAI of the novel, microsphere-based, extended-release formation of triamcinolone acetonide leads to a prolonged reduction in symptoms of osteoarthritis [16]. Deducted from studies above, IAI of analgesic cocktail may also play a role in pain relief after TKA. In addition, PAI could increase the risk of paralysis of common peroneal nerve, while IAI may consume less operation time and have no increased risks. Therefore, although most surgeons perform LIA in TKA as PAI, and never just IAI, we are curious about the comparison within LIA administration techniques, between PAI and IAI. In 2015, Perret published an article comparing PAI and IAI in TKA in Australia [17]. The study failed to show statistically significant benefit in either technique. Besides, the study is not a prospective randomized controlled trial (RCT). At present, there is no RCT existing towards the comparison between PAI and IAI of analgesic cocktail in TKA.

This randomized study aimed at determining the effect of administration techniques of LIA on pain relief and postoperative rehabilitation. We compared analgesics efficacy and safety of PAI versus IAI in patients receiving simultaneous bilateral TKA during the in-hospital period.

## Methods

### Trial design and ethics approval

This single-centre, prospective randomized controlled trial (RCT) was performed at the Department of Orthopedic Surgery, Peking Union Medical College Hospital, following the Consolidated Standards of Reporting Trials (CONSORT) statement guidelines for reporting parallel-group randomized controlled trial [18]. The eligible patients were supposed to receive simultaneous bilateral total knee arthroplasty, in which one side of the knees underwent PAI and another one underwent IAI. The details of randomized allocation were described in the following ‘Randomization and Blinding’ part. The study was approved by the institutional review board of Peking Union Medical College Hospital (25th Oct, 2016) and performed in accordance with the standards of 1964 Declaration signed in Helsinki. All patients participating in this trial signed informed consent. The trial was registered on Chinese Clinical Trial Registry as ChiCTR1800020420 (respectively registered on 29th December, 2018).

### Eligibility

Patients were identified on the day before scheduled surgery and evaluated for eligibility. Patients will be enrolled in the study if they meet the criteria: 1) older 18 years old; 2) receive simultaneous bilateral total knee arthroplasty during the same anaesthesia session; 3) diagnosed with osteoarthritis or rheumatoid arthritis. Exclusion criteria are:1) a history of allergy to any of the injectable drug ingredients or excipients; 3) severe deformity of genu varum or valgum (change of femoral-tibial angle > 20°); 4) comorbid with bronchospasm, acute rhinitis, nasal polyps, angioneurotic edema, urticaria, and other allergic reactions after taking aspirin or NSAIDs (including COX-2 inhibitors); 5) severe liver injury (serum albumin< 25 g/L or Child-Pugh score ≥ 10), inflammatory bowel disease, opioids abuse, a body mass index (BMI) of > 35 kg/m^2^; 6) American Society of Anesthesiologists (ASA) category of > 3, or physical, emotional, or neurological conditions that would compromise compliance with postoperative rehabilitation and assessment.

### Randomization and blinding

The LIA administration technique and the order of the operations for the two knees of each participant were randomly allocated using a computer-generated table, which was conducted by investigators not involving in the whole trial protocol except for this randomization and blinding procedure. For each participant, a sealed envelope was opened in the operating room to identify the treatment assignment. The patient received PAI on one side and IAI on another. The orthopaedic surgeon was informed about the administration allocation before skin incision. The patients, data collectors, and analysts were blinded during the entire trial.

### Interventions procedure

All the surgeries were performed through medial parapatellar approach by the corresponding author (Xisheng Weng) with 250 mmHg tourniquet under general anaesthesia. The constituent of administered cocktail solution in our study combined the components in previous studies [19–22], consisting of 200 mg ropivacaine, 100μg fentanyl, 0.25 mg adrenaline, 50 mg flurbiprofen axetil, and 1 mg diprospan, with addition of normal saline to a 60 mL soliton. A drainage tube was placed laterally to the prosthesis components in every joint, clamped for 3 h [23] and then unlocked, and removed in the second morning after surgery. The drainage tube has 6 orifices and all of them were located inside the articular cavity.

Intervention procedure was conducted according to the randomized allocation. In PAI group, before prosthesis installation, 20 mL of cocktail solution was injected into the posterior capsule, including femoral attachments of anterior cruciate ligament and posterior cruciate ligament, posteromedial and posterolateral capsules. After prosthesis installation, the residual 40 mL was injected into the medial and lateral collateral ligament, quadriceps tendon, patellar tendon, pes anserinus, fat pad and subcutaneous tissues. In IAI group, after closure of deep fascia, the cocktail solution was injected into the articular cavity through the drainage tube. It is the watertight test that we perform after suturing the deep fascia in every joint to check the watertight condition of the area. If fluids were leaking in somewhere, we would make more sutures to ensure the articular cavity was watertight. Both PAI and IAI were single-shot administrations. No participants received any regional nerve blocks or epidural block during the whole perioperative period. Participants were free to choose the use of PCA according to their wills.

After surgery, participants routinely received 40 mg of parecoxib in every 12 h and 650 mg of acetaminophen in every 8 h. The rescue analgesia treatment included morphine, oxycodone or pethidine. The consumption of overall opioids of every participant was documented.

### Outcome measurements

The primary outcome was pain intensity at rest or during activity assessed by NRS pain score at 3, 6, 12, 24, 36, 48, and 72 h after surgery. Secondary outcome included active and passive range of motion at 1, 2 and 3 days after surgery, volume of wound drainage, postoperative days required to perform straight leg raise, length of hospital stay and opioids use in morphine equivalents. Range of motion (ROM) was calculated as the sum of angles of knee flexion and extension measured by a long-arm goniometer without removing outside dressing. In our study, active ROM means patients bend their knee joints freely without enforcement, and passive ROM means investigators bend their knee joints as most under their tolerance. The operation time was counted from skin incision to wound dressing. Morphine consumption was calculated as the sum of morphine equivalents divided by the weight of the patient.

### Sample size

Our hypothesis was to substantiate the non-inferiority of IAI compared with PAI. The sample size was calculated according to the following formula [24]:

*n* = 2*[(u_1-α/2_ + u_1-β_) σ/δ]^2^.

To show a clinically important difference of 1.3 [25] in NRS pain score between PAI group and IAI group, with a standard deviation of 2.0 according to the published article [17], a power 0.90 and a two-tailed significance of <0.05, each group required 49 subjects.

### Statistical analysis

Measurement data were expressed as mean and 95% confidence interval (95% CI). Shapiro–Wilk test and Levene test were performed to evaluate normality and homogeneity of variance of the data, respectively. If data did not comply with normal distribution or equal variance, a non-parametric test (Mann-Whitney) was applied; if else, student t-test was undertaken to analyse the difference between the two groups. The dichotomous data were analysed by Fisher’s exact test, in that 50% of cells have expected count less than 5. SPSS version 25.0 software was used during the analysis process.

## Results

### Baseline characteristics

**Fig. 1:**
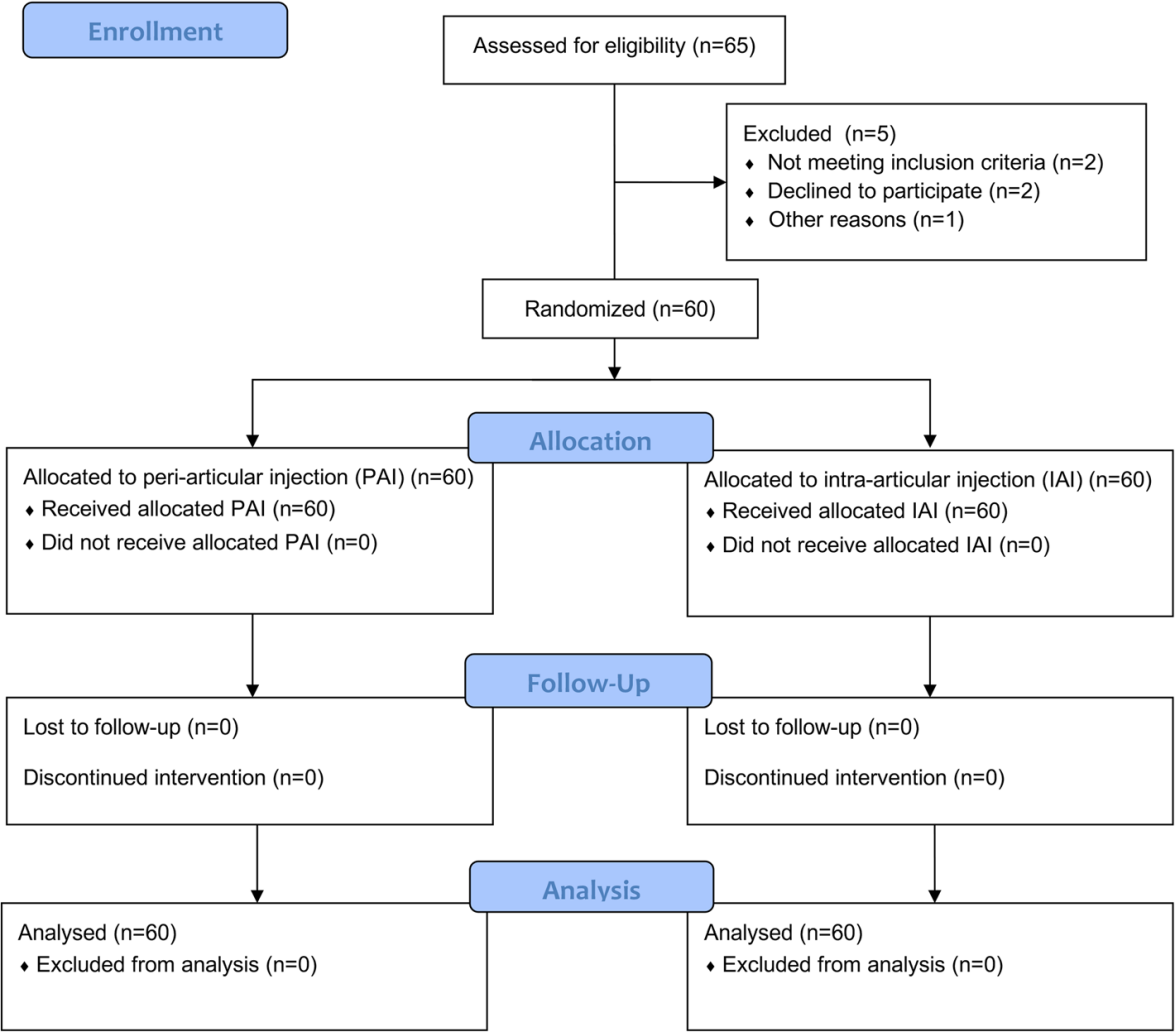
Enrollment, Allocation, Follow-up and Analysis of the Study

Between February 2017 and July 2018, 65 patients were enrolled in the study, among which 5 patients were excluded for violating criteria (severe deformity with more than 5 mm bone defect of tibia plateau inspected during surgery, refusal to participate and incoordination to respond) (Fig. 1). A total of 60 patients participated in the study. All of them finished the process of randomization, allocation, trial administration and postoperative assessment. Baseline characteristics of the participants are illustrated in Table 1, including gender, age, body mass index, ethnics, diagnosis, and ASA grade. There were no differences in NRS pain score and ROM between two groups before the surgery and intervention.

**Table 1:** Baseline Characteristics of the Patients*

Characteristic	Peri-articular Injection	Intra-articular Injection	*P* value
Female, n (%)	55 (91.6)		
Age—yr mean [95%CI]	65.8 [64.0, 67.6]		
Body mass index† − -kg/m^2^ mean [95%CI]	27.7 [26.7, 28.8]		
Ethnics, n (%)			
Han	56 (93.3)		
Minority‡	4 (6.7)		
Diagnosis, n (%)			
Osteoarthritis	56 (93.3)		
Rheumatoid arthritis	4 (6.7)		
ASA grade, n (%)			
I	3 (5.0)		
II	53 (88.3)		
III	4 (6.7)		
IV	0		
Numerical rating scale at rest mean [95%CI]	0.16 [0.04, 0.28]	0.20 [0.04, 0.35]	0.855
Numerical rating scale during activity mean [95%CI]	5.25 [4.87, 5.62]	4.98 [4.51, 5.45]	0.317
Range of motion actively§ mean [95%CI]	94.8 [93.3, 96.2]	94.0 [92.7, 95.4]	0.453
Range of motion passively ¶ mean [95%CI]	115.9 [114.2, 117.5]	114.0 [112.4, 115.6]	0.103

* No significant differences between groups in the reported characteristics were found at baseline

† The body-mass index is the weight in kilograms divided by the square of the height in meters

‡ Four patients are Chinese minorities, including two Manchu and two Mongols

§ Range of motion actively is patients bending knees by themselves

¶ Range of motion passively is physicians bending patients’ knees

### Primary outcome

**Fig. 2:**
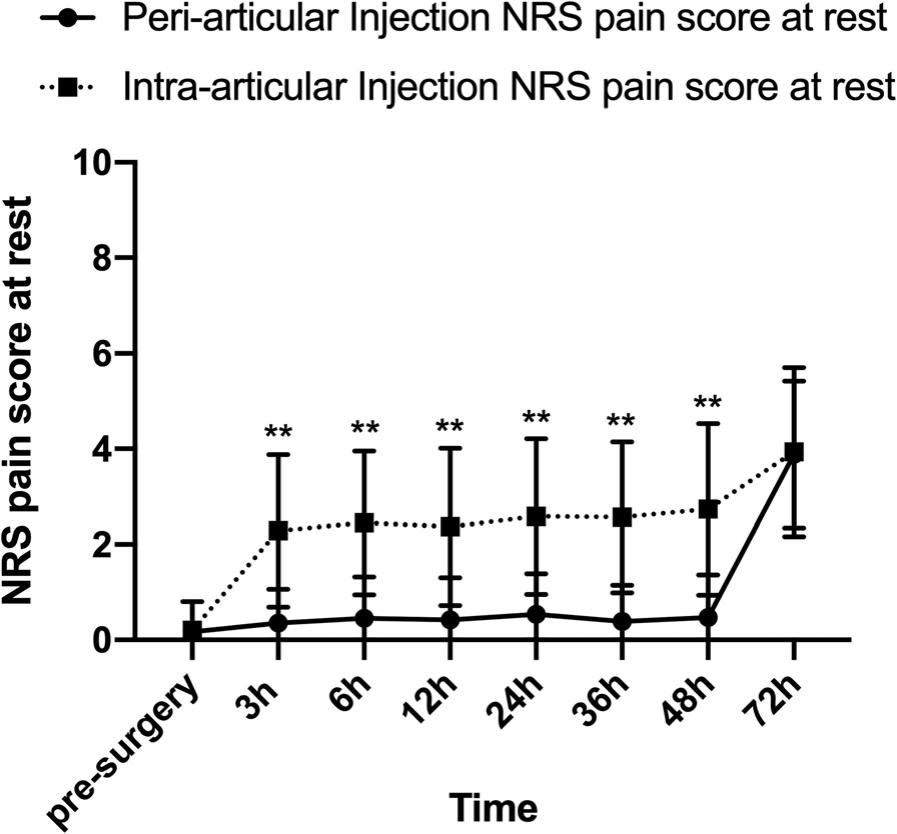
VAS pain score at rest. *P < 0.05, **P < 0.01

**Fig. 3:**
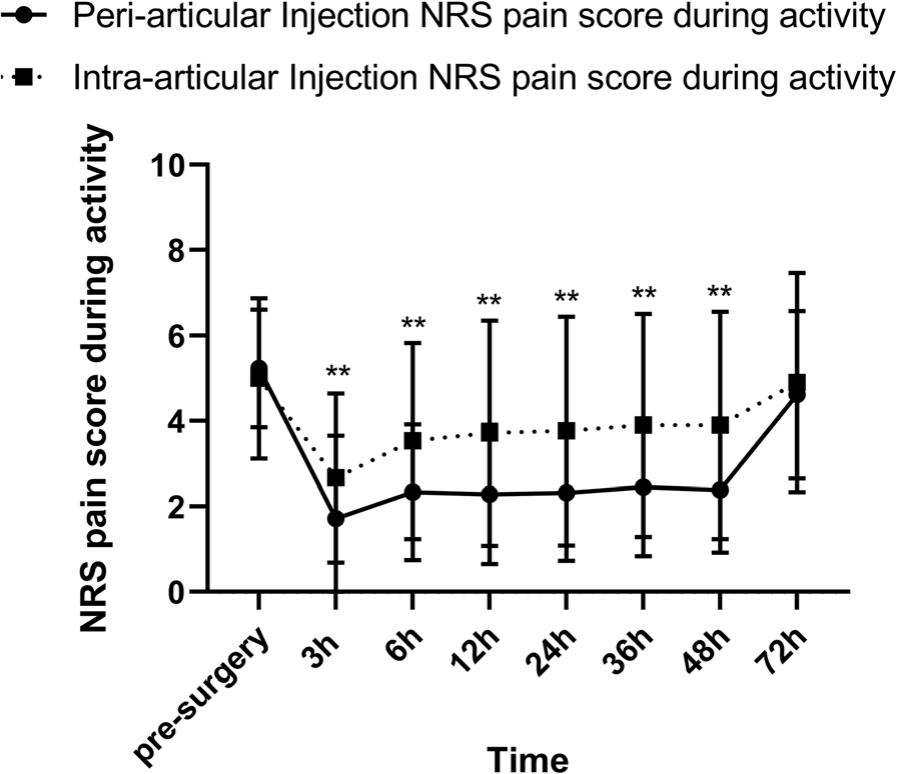
VAS pain score during activity. *P < 0.05, **P < 0.01

During the first 48 h after surgery, NRS pain score in PAI group was significantly lower than that in IAI group (Fig. 2, Fig. 3 and Additional file 1: Table S1). The difference of NRS pain score between the two groups was larger at rest compared with that during activity. The differences of NRS pain score at 3 h, 6 h, 12 h, 24 h, 36 h, 48 h at rest and at12h, 24 h, 36 h and 48 h during activity were over 1.3 with a clinically important difference. The largest difference in NRS pain score occurred in 48 h after surgery at rest (PAI: 0.68 [0.37, 0.98]; IAI: 2.63 [2.16, 3.09], *P* < 0.001; Between-group difference: − 1.95 [− 2.50, − 1.39]) or during activity (PAI: 2.46 [2.07, 2.85]; IAI: 3.90 [3.27, 4.52], *P* = 0.001; Between-group difference in change: − 1.43 [− 2.16, − 0.70]). There were no differences between two groups in NRS pain score at 72 h after the surgery at rest (*P* = 0.426) or during activity (*P* = 0.287).

### Secondary outcome

**Fig. 4:**
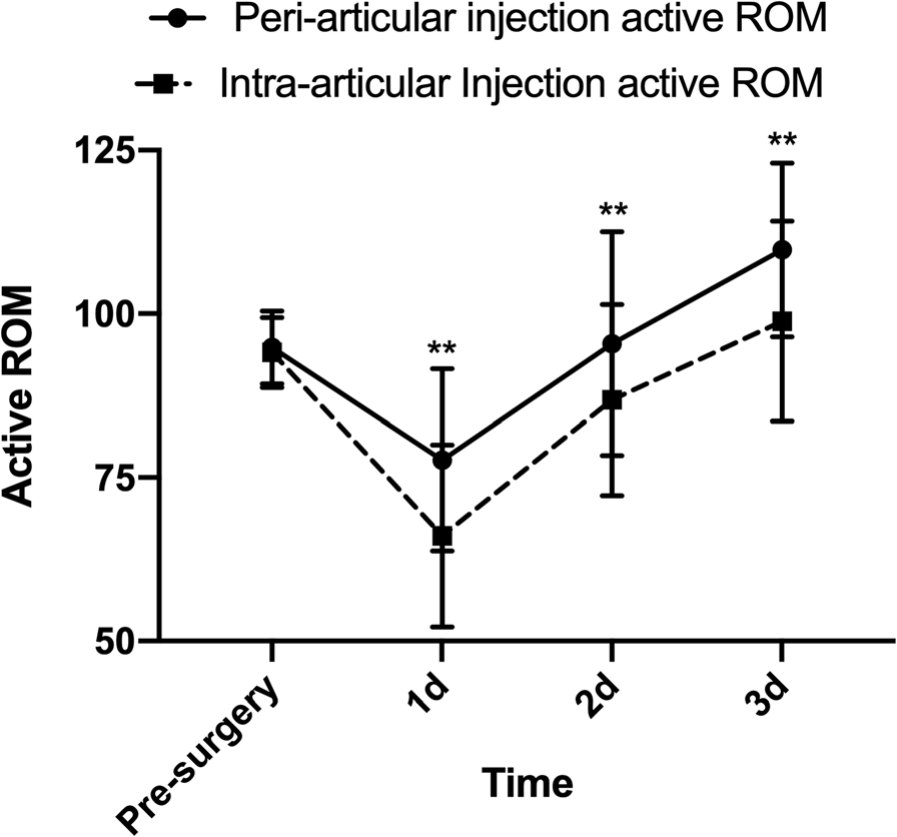
Active ROM. *P < 0.05, **P < 0.01

**Fig. 5:**
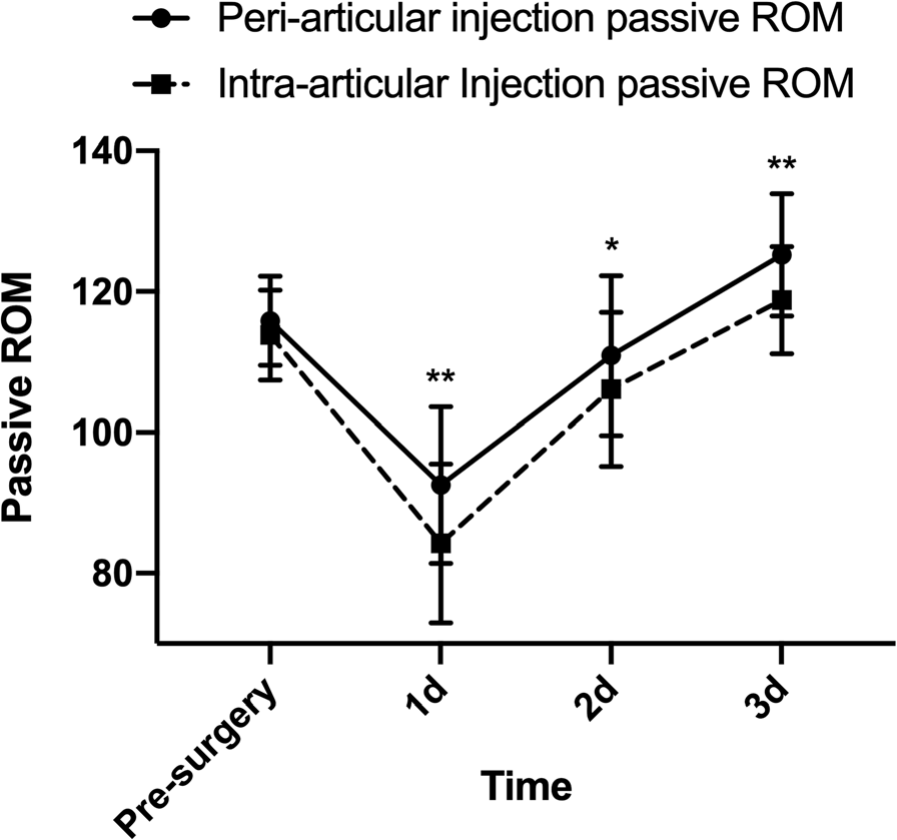
Passive ROM. *P < 0.05, **P < 0.01

PAI group had better results of active ROM and passive ROM in the first 3 days after surgery compared with IAI group (Fig. 4, Fig. 5, and Additional file 2: Table S2). The largest difference in active ROM (PAI: 77.6 [74.0, 81.2]; IAI: 66.0 [62.4, 69.6], *P* < 0.001; Between-group difference in change: 11.5 [6.5, 16.6]) and passive ROM (PAI: 91.7 [88.8, 94.7]; IAI: 84.9 [82.0, 87.9], *P* = 0.001; Between-group difference in change: 6.8 [2.6, 10.9]) between two groups took place at day 1 after surgery. There were no significant differences in operation time (*P* = 0.614) and wound drainage volume (*P* = 0.607) (Table 2). PAI group consumed less time to perform straight leg raise postoperatively (PAI: 1.08 [0.90, 1.25]; IAI: 1.45 [1.21, 1.68], *P* = 0.012; Between-group difference in change: − 0.36 [− 0.65, − 0.08]). The length of hospital day was 5.53 [4.98, 6.07] and morphine consumption was 1.23 mg/kg [1.15, 1.31].

**Table 2:** Secondary Outcomes

Characteristic	Mean [95%CI]			*P* Value
	Peri-Articular Injection	Intra-Articular Injection	Between-Group Difference in Change [95%CI]	
Operation time (min)	70.0 [68.4, 71.6]	69.8 [67.9, 71.7]	0.2 [−2.2, 2.6]	0.614
Wound drainage volume day 1 (mL)	85.7 [66.1, 105.4]	82.8 [65.1, 100.4]	2.9 [− 23.1, 29.1]	0.992
Wound drainage volume day 2 (mL)	95.9 [74.3, 117.5]	87.6 [68.3, 107.0]	8.2 [−20.4, 36.9]	0.731
Wound drainage volume in total (mL)	181.7 [148.5, 214.9]	170.4 [138.0, 202.8]	11.2 [−34.6, 57.1]	0.607
Postoperative days required to perform straight leg raise	1.08 [0.90, 1.25]	1.45 [1.21, 1.68]	−0.36 [−0.65, −0.08]	0.026
Morphine consumption (mg/kg)	1.23 [1.15, 1.31]		–	–
Length of hospital stay	5.53 [4.98, 6.07]		–	–

### Complication

In PAI group, there was one case complicated with deep venous thrombus, one with nerve palsy and one with fat liquefaction. In IAI group, there was one case complicated with deep venous thrombus. Generally, there were no differences in wound complications between the two groups (Table 3). The overall wound complication rate was 3/60 in PAI group and 1/60 in IAI group (Relative risk, 1.526 [0.842, 2.768], *P* = 0.619).

**Table 3:** Wound Complications

Complications	Peri-Articular Injection, n (%)	Intra-Articular Injection, n (%)	Relative risk of PAI [95% CI]	*P* value
Deep venous thrombus	1 (1.6)	1 (1.6)	1.000 [0.247, 4.045]	1.000
Nerve palsy	1 (1.6)	0	2.017 [1.683.2.418]	1.000
Fat liquefaction	1 (1.6)	0	2.017 [1.683.2.418]	1.000
Overall infection	0	0	–	–
Articular hematoma	0	0	–	–
Overall complications	3 (5.0)	1 (1.6)	1.526 [0.842, 2.768]	0.619

## Discussion

Our results demonstrate that PAI provides superior analgesic benefit to IAI in patients receiving TKA. The advantage of PAI over IAI on NRS pain score faded off after 48 h, while ROM was continuously better in PAI group than IAI group during the first 3 days after the surgery. In addition, it took less time for PAI group to perform straight leg raise postoperatively. There were no differences in operation time, volume of wound drainage and wound complications between two groups. Our study substantiated the superiority of PAI to IAI in analgesia after total knee arthroplasty. Therefore, PAI technique was recommended for performing LIA in TKA.

PAI group showed a statistically significant reduction in postoperative VAS pain scores in a previous study [17], which positively correlated with NRS pain scores in our study [26]. In a retrospective study [27], Tietje demonstrated that patients receiving PAI of local anaesthetics in TKA had a noticeable decrease in length of hospital stay and incidence of postoperative nausea and vomiting when compared to patients receiving IAI. In the early period after surgery, it is pain that mainly accounts for patients hospitalization [2]. Besides, the occurrence of nausea and vomiting in patients after surgery may vary from the usage of opioids [7]. Therefore, it could be deducted from the results of Tietje that the analgesic benefit of PAI may underlie the decreased length of hospital stay and incidence of postoperative nausea and vomiting. In the current study, PAI had advantages of pain relief over IAI, corresponding with our deduction from Tietje study.

There are several mechanisms underlying the analgesic benefit of PAI over IAI. According to a previous cadaveric study [28], the outer capsule is more abundant of innervation such as saphenous nerve and genicular nerves, while the inner synovium and articular cavity have fewer nerve distribution. Another histologic survey of human cadaveric knees performed by Jiranek et al. [29] elucidated the distribution of free nerve endings after hematoxylin and eosin staining. High concentrations of nociceptors were found in the medial and lateral retinacula, patellar tendon, pes anserinus, and meniscofemoral ligaments. The lowest concentration was seen in the central portion of the anterior cruciate ligament. Thus, the conduct of PAI could be more effective than IAI because of denser innervation of the outside capsule and soft tissues in the knee joint. Besides, since we placed a drainage tube in every joint, solution in the articular cavity was more likely to be drained out and solution in the soft tissues around the knee joint could continue to work out. It would be more difficult for cocktail solution of PAI group to escape from the joint than that of IAI. It also might be the persistent effect of cocktail solution in PAI group that contributes to the analgesic benefits. The volume of cocktail solution was the same in both groups, and according to our previous assumptions, the volume of wound drainage of IAI group was supposed to outnumber that of PAI group. However, there was no difference in the volume of wound drainage in our study. This paradox requires more substantive evidence to explain. For the further investigation to uncover the potential mechanism, a biocompatible and undegraded detector could be included in the cocktail solution to detect the real-time concentration and volume of the solution constituents in the articular cavity and soft tissues around the knee joint.

To our knowledge, this is the first RCT study comparing analgesic efficacy and safety of PAI with that of IAI in patients receiving simultaneous bilateral TKA. The highlight of our study is the self-control design, where participants received PAI on one side and IAI on another. Owing to the homogeneity inside one participant, the only possible explanation for the remarkable differences in outcomes may lay in distinctive interventions. The conclusion of our study is confirmative.

However, there is no exception for limitations in our study. Firstly, the ceiling effect makes it impossible to distinguish the differences in systemic adverse effects, ambulation mobility and morphine consumption between two groups. In addition, one pain could increase or reduce the other. Thus, the difference in our study could be overestimated or underestimated. Despite the qualitative conclusion in the study, further research is required to determine the exact difference between the two groups. Besides, the outcomes were only limited to in-hospital data without long-term follow-up data and the long-term effect needs to be further evaluated.

## Conclusion

Generally, we conducted a randomized controlled trial to compare the analgesic efficacy and safety of PAI versus IAI in patients receiving simultaneous total knee arthroplasty. PAI had more analgesic benefits than IAI after the surgery. There were no differences between PAI and IAI in wound drainage, operation time, and wound complications. The administration technique of PAI is recommended when performing LIA in TKA.

## Supplementary information

**Additional file 1: Table S1.** Numerical Rating Scale (NRS) at rest or during activity

**Additional file 2: Table S2.** Range of Motion

## Data Availability

The datasets used during the current study are available from the corresponding author on reasonable request.

## Abbreviations

ASA: American Society of Anesthesiologists
BMI: Body mass index
CI: Confidence interval
CONSORT: Consolidated standards of reporting trials
IAI: Intra-articular injection
LIA: Local infiltration analgesia
NRS: Numerical rating scale
NSAIDs: Nonsteroidal anti-inflammatory drugs
PAI: Peri-articular injection
PCA: Patient-controlled analgesia
RCT: Randomized controlled trial
ROM: Range of motion
TKA: Total knee arthroplasty
