# Supplementary material for: The analgesic efficacy and safety of peri-articular injection versus intra-articular injection in one-stage bilateral total knee arthroplasty: a randomized controlled trial
Source: BMC Anesthesiol. 2020 Jan 4;20:2. doi: 10.1186/s12871-019-0922-4 (PMC6942284; doi:10.1186/s12871-019-0922-4)
Supplement: Supplementary file 1 — Additional file 1: Table S1. Numerical Rating Scale (NRS) at rest or during activity [file 12871_2019_922_MOESM1_ESM.docx]

**Supplementary Table 1 Numerical Rating Scale (NRS) at rest or during activity**

| Characteristic | Mean [95%CI] | | | P Value |
| --- | --- | --- | --- | --- |
|  | Peri-Articular Injection | Intra-Articular Injection | Between-Group Difference in Change [95%CI] |  |
| NRS at rest 3h | 0.51 [0.26, 0.77] | 2.01 [1.60, 2.42] | -1.50 [-1.97, -1.02] | <0.001 |
| NRS at rest 6h | 0.61 [0.34, 0.89] | 2.45 [2.01, 2.88] | -1.83 [-2.33, -1.32] | <0.001 |
| NRS at rest 12h | 0.58 [0.30, 0.86] | 2.23 [1.83, 2.63] | -1.65 [-2.13, -1.16] | <0.001 |
| NRS at rest 24h | 0.68 [0.42, 0.94] | 2.45 [2.02, 2.87] | -1.76 [-2.26, -1.27] | <0.001 |
| NRS at rest 36h | 0.53 [0.30, 0.76] | 2.45 [2.01, 2.88] | -1.91 [-2.40, -1.42] | <0.001 |
| NRS at rest 48h | 0.68 [0.37, 0.98] | 2.63 [2.16, 3.09] | -1.95 [-2.50, -1.39] | <0.001 |
| NRS at rest 72h | 3.60 [3.21, 3.98] | 3.80 [3.34, 4.25] | -0.20 [-0.79, 0.39] | 0.426 |
| NRS during activity 3h | 1.81 [1.31, 2.32] | 2.70 [2.21, 3.18] | -0.88 [-1.57, -0.18] | 0.006 |
| NRS during activity 6h | 2.40 [1.99, 2.80] | 3.56 [2.98, 4.14] | -1.16 [-1.87, -0.46] | 0.004 |
| NRS during activity 12h | 2.40 [1.97, 2.82] | 3.76 [3.09, 4.43] | -1.36 [-2.15, -0.57] | 0.004 |
| NRS during activity 24h | 2.38 [1.99, 2.77] | 3.70 [3.07, 4.32] | -1.31 [-2.04, -0.58] | 0.003 |
| NRS during activity 36h | 2.51 [2.09, 2.94] | 3.85 [3.22, 4.47] | -1.33 [-2.08, -0.58] | 0.003 |
| NRS during activity 48h | 2.46 [2.07, 2.85] | 3.90 [3.27, 4.52] | -1.43 [-2.16, -0.70] | 0.001 |
| NRS during activity 72h | 4.58 [4.13, 5.03] | 4.96 [4.38, 5.54] | 0.36 [-1.10, 0.34] | 0.287 |
