# Supplementary material for: The analgesic efficacy and safety of peri-articular injection versus intra-articular injection in one-stage bilateral total knee arthroplasty: a randomized controlled trial
Source: BMC Anesthesiol. 2020 Jan 4;20:2. doi: 10.1186/s12871-019-0922-4 (PMC6942284; doi:10.1186/s12871-019-0922-4)
Supplement: Supplementary file 2 — Additional file 2: Table S2. Range of Motion [file 12871_2019_922_MOESM2_ESM.docx]

**Supplementary Table 2 Range of Motion**

| Characteristic | Mean [95%CI] | | | P Value |
| --- | --- | --- | --- | --- |
|  | Peri-Articular Injection | Intra-Articular Injection | Between-Group Difference in Change [95%CI] |  |
| Active ROM day 1 | 77.6 [74.0, 81.2] | 66.0 [62.4, 69.6] | 11.5 [6.5, 16.6] | <0.001 |
| Active ROM day 2 | 95.4 [91.0, 99.8] | 86.8 [83.0, 90.6] | 8.5 [2.8, 14.3] | 0.001 |
| Active ROM day 3 | 109.7 [106.3, 113.1] | 98.8 [94.9, 102.8] | 10.8 [5.7, 16.0] | <0.001 |
| Passive ROM day 1 | 91.7 [88.8, 94.7] | 84.9 [82.0, 87.9] | 6.8 [2.6, 10.9] | 0.001 |
| Passive ROM day 2 | 111.5 [108.9, 114.1] | 106.2 [103.3, 109.1] | 5.3 [1.3, 9.2] | 0.035 |
| Passive ROM day 3 | 124.7 [122.4, 127.0] | 119.2 [117.2, 121.2] | 5.4 [2.4, 8.5] | <0.001 |
